# Physiological Importance of Pectin Modifying Genes During Rice Pollen Development

**DOI:** 10.3390/ijms21144840

**Published:** 2020-07-08

**Authors:** Yu-Jin Kim, Ho Young Jeong, Seung-Yeon Kang, Jeniffer Silva, Eui-Jung Kim, Soon Ki Park, Ki-Hong Jung, Chanhui Lee

**Affiliations:** 1 Graduate School of Biotechnology & Crop Biotech Institute, Kyung Hee University, Yongin 17104, Korea; yujinkim@khu.ac.kr (Y.-J.K.); moss7894@naver.com (S.-Y.K.); jeniffersilva.yat@gmail.com (J.S.); alice804@khu.ac.kr (E.-J.K.); 2 Department of Plant & Environmental New Resources, College of Life Sciences, Kyung Hee University, Yongin 17104, Korea; ratank@khu.ac.kr; 3 School of Applied Biosciences, Kyungpook National University, Daegu 41566, Korea; psk@knu.ac.kr

**Keywords:** rice, pollen, pectin, pectin methylesterase, pectin methylesterase inhibitor, pollen tube growth

## Abstract

Although cell wall dynamics, particularly modification of homogalacturonan (HGA, a major component of pectin) during pollen tube growth, have been extensively studied in dicot plants, little is known about how modification of the pollen tube cell wall regulates growth in monocot plants. In this study, we assessed the role of HGA modification during elongation of the rice pollen tube by adding a pectin methylesterase (PME) enzyme or a PME-inhibiting catechin extract (Polyphenon 60) to in vitro germination medium. Both treatments led to a severe decrease in the pollen germination rate and elongation. Furthermore, using monoclonal antibodies toward methyl-esterified and de-esterified HGA epitopes, it was found that exogenous treatment of PME and Polyphenon 60 resulted in the disruption of the distribution patterns of low- and high-methylesterified pectins upon pollen germination and during pollen tube elongation. Eleven PMEs and 13 PME inhibitors (PMEIs) were identified by publicly available transcriptome datasets and their specific expression was validated by qRT-PCR. Enzyme activity assays and subcellular localization using a heterologous expression system in tobacco leaves demonstrated that some of the pollen-specific PMEs and PMEIs possessed distinct enzymatic activities and targeted either the cell wall or other compartments. Taken together, our findings are the first line of evidence showing the essentiality of HGA methyl-esterification status during the germination and elongation of pollen tubes in rice, which is primarily governed by the fine-tuning of PME and PMEI activities.

## 1. Introduction

All plant cells are encased by walls with a distinct polysaccharide composition. Some specialized cells, including those of root hairs and pollen tubes, undergo rapid directional elongation [1,2,3]. Once differentiated, these types of cells depend upon the coordinated action of cell wall-modifying enzymes (CWMEs), which promote wall loosening and extensibility by disrupting chemical bonds or selectively removing specific polysaccharide side chains [4]. All higher plants contain a wide range of CWMEs, such as expansin, glycosyl hydrolases, and carbohydrate esterases and lyases [5,6].

Pectin is a polysaccharide found mainly in the middle lamella and primary cell wall, and plays a critical role in wall plasticity and cellular adhesion [7]. Compositional analysis of grass cell walls has revealed that pectin accounts for approximately 5% of the cell wall components [8,9]. Pectin has a galacturonic acid (GalUA)-rich backbone of four domains that can be distinguished based on their structure and substitution patterns: homogalacturonan (HGA), rhamnogalacturonan I (RGI), rhamnogalacturonan II (RGII), and xylogalacturonan (XGA) [6,9]. HGA, a linear polymer of (1,4)-linked-α-GalUA, is the predominant form of pectin and becomes heavily methyl-esterified at the C-6 carboxyl by pectin methyltransferases when in the Golgi apparatus [10]. Following this, it is transported and deposited into cell walls to connect to their matrices, wherein it undergoes further modifications depending on the cell type and its position in the pectin network. One of the best known types of modification is demethyl-esterification (selective removal of methyl groups) by pectin methylesterases (PMEs), which is believed to profoundly impact the wall’s mechanical properties in a variety ways. Thus, fine-tuning PME activities are imperative for fulfilling cellular functions.

PME proteins are grouped into two types, depending on the presence of an N-terminal pro region, and labeled as type 1 (with pro region) and type 2 (without pro region) [11,12]. This N-terminal pro region appears to function as a self-inhibitory domain in the Golgi apparatus, which is removed proteolytically before being transported out by secretory vesicles [13]. In addition, the enzymatic action of PME is regulated by PME inhibitors (PMEIs), which belong to a large multigene-encoded protein family. Co-crystallization experiments using the tomato (*Solanum lycoperscicum*) PME and kiwi (*Actinidia chinensis*) PMEI complex have demonstrated that PMEIs bind specifically to the pectin-binding cleft of PME [14], and as such, it is generally believed that specific PME-PMEI pairs exist. A recent simulation analysis using *Arabidopsis* PME (AtPME3) and PMEIs (AtPMEI4 and AtPMEI9), which are coexpressed in roots, uncovered key residues playing a critical role in the specificity and pH-dependence of inhibitor binding [15].

Given that all higher plants possess PMEs and PMEIs, and that pectin HGA is found in most plant cell types, it is not surprising that misregulation of those genes can cause a wide range of developmental abnormalities. Changes in the transcription levels of specific PMEs and PMEIs, either through overexpression or knock-out approaches, strongly influence the degree of pectin methyl-esterification in certain cell types and developmental processes, including dark-grown hypocotyl elongation, pollen tube elongation, dormancy, germination, seed mucilage production and pathogenesis [16,17]. Importantly, several PME and PMEI isoforms show specific expression in pollen and pollen tubes in dicot plants, and *Arabidopsis* mutants with reduced PME activities have displayed severe defects in pollen tube growth and morphology [18]. Furthermore, adding PME or pectinase to a pollen germination medium resulted in abnormal growth of the pollen tube in *Solanum chacoense* [19]. Immunohistochemical studies in dicot plants have revealed that highly methyl-esterified HGA is prevalent in the apex of the pollen tube, whereas minimally methyl-esterified HGA is dominant in lateral region of the pollen tube [10]. The establishment of this disproportion is governed by the fine-tuning of PMEIs.

Previously, we reported that the rice genome has 43 PMEs and 49 PMEIs [7,20]. Although it is well known that dynamic changes in the methyl-esterification of HGA by PME and PMEI enzymes are critical for proper pollen tube growth in dicot plants, the involvement of PME and PMEI in regulating monocot pollen tube development is still poorly understood. To investigate their importance in the development of rice pollen, we first assessed the effects of pollen tube growth following the addition of a PME enzyme and PME-inhibiting catechin extract (Polyphenon 60) to an in vitro pollen germination medium. Eleven pollen-specific PMEs and 13 PMEIs were identified in rice and enzymatic activities were measured. Our findings showed that spatial, post-translational control of PME activities by PMEI plays a key role in disproportional deposition of methyl-esterified HGA in the pollen tube walls of rice.

## 2. Results

### 2.1. The Effects of a PME and PME-Inhibiting Catechin Extract on In Vitro Pollen Germination in Rice

**Figure 1:**
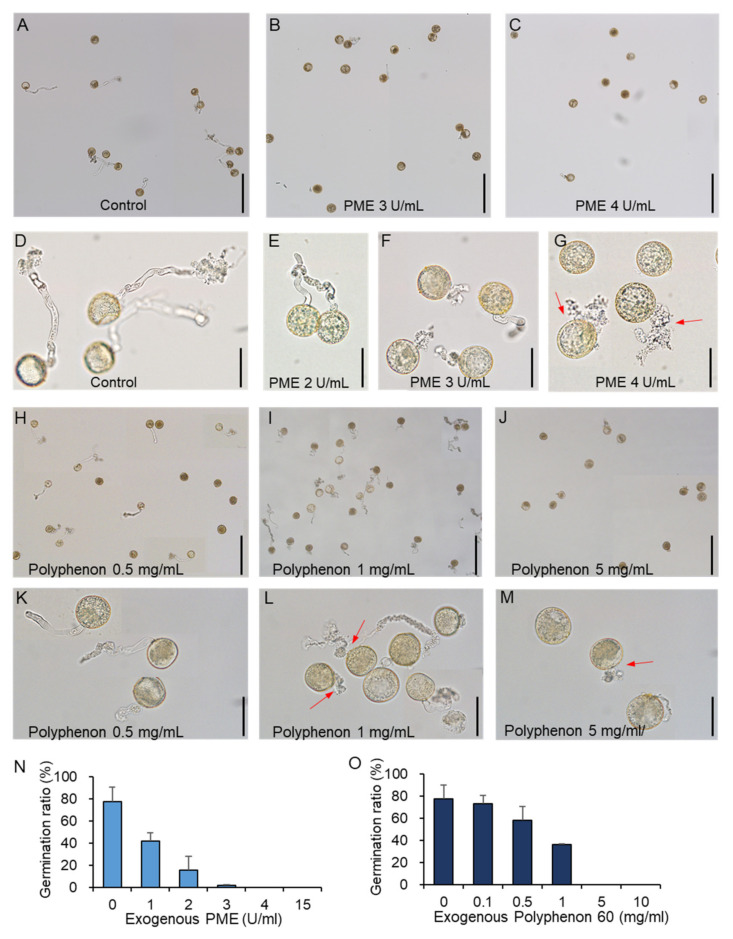
The effect of PME and Polyphenon 60 treatments on rice pollen germination and tube growth. In vitro pollen germination of rice pollen were analyzed on the liquid germination media without (**A**,**D**) and with exogenous PME (**B**,**C**,**E**–**G**) and Polyphenon 60 (**H**–**M**). The concentration was indicated in each image. Red arrows indicate pollen grains which rupture early without forming intact pollen tubes. Bars = 200 µm (**A**–**C**,**H**–**J**) and 50 µm (**D**–**G**,**K**–**M**). (**N**,**O**) The percentage of germinated pollen grain with an intact tube was presented. Red arrow marks pollen grains with burst or disintegrated tubes. Three independent experiments + SD (*n* > 100).

Previous genetic and biochemical studies on dicot pollen development have shown that the degree of HGA methyl-esterification is one of the most critical determinants of pollen germination and tip elongation [3,11,21,22,23]. To directly determine if fine-tuning of HGA methyl-esterification status via PME and PMEI enzymatic activities is essential during rice pollen tube growth, we added various concentrations of commercial PME to an in vitro pollen germination medium, with the intention of disturbing the cellular balance and creating minimally methyl-esterified HGA across the pollen cell walls. In our experiment, about 80% of the rice pollens germinated within 20 min (Figure 1A,D), and a decreased rate of pollen germination was observed in the PME supplemented medium (Figure 1B,C). In addition, pollen tube growth was severely affected: while normal rice pollen tubes grew to an average length of 100 µm, the germinated pollen tubes ceased to grow beyond 50 µm in length at low concentrations of PME (Figure 1D–F), and burst immediately at the highest concentration of PME (Figure 1G, marked by red arrow). Nearly 50% of the pollen grains germinated at a concentration of 1 units·mL^−1^ PME, nearly 2% germinated at a concentration of 3 units·mL^−1^ PME, and no pollen germination was observed at higher concentrations of 4–15 unit·mL^−1^ PME (Figure 1N). To further verify that the degree of HGA methyl-esterification was a critical factor for pollen tube growth, a PME-inhibiting catechin extract (Polyphenon 60) was added to the germination medium [24,25]. Although epigallocatechin-3-gallate, the main component of Polyphenon 60, has been recently reported to affect broad ranges of plant physiology including regulation of jasmonic acid signaling and disease resistance, its specific inhibitory effect to PME has been well documented [26]. The amount of germination was slightly affected by the concentration of 0.5 mg·mL^−1^ of the catechin extract (Figure 1H), and decreased in a dose-dependent manner (Figure 1H–J,O). Germination of the pollen tube decreased significantly following treatment with high concentrations of Polyphenon 60, compared to that of the control (Figure 1I,J). In addition, concentrations greater than 1 mg·mL^−1^ damaged the integrity of the pollen tube walls and inhibited elongation, inducing an early burst (Figure 1L,M, marked by red arrow). Our findings suggest that the plasticity and integrity of cell walls during pollen tube growth are primarily governed by the fine-tuning of PME and PMEI activities, which is one of the critical factors for pollen germination and elongation.

### 2.2. Alteration of Immunolabeling Patterns of Pectins by Exogenous Treatment of PME and Polyphenon 60

**Figure 2:**
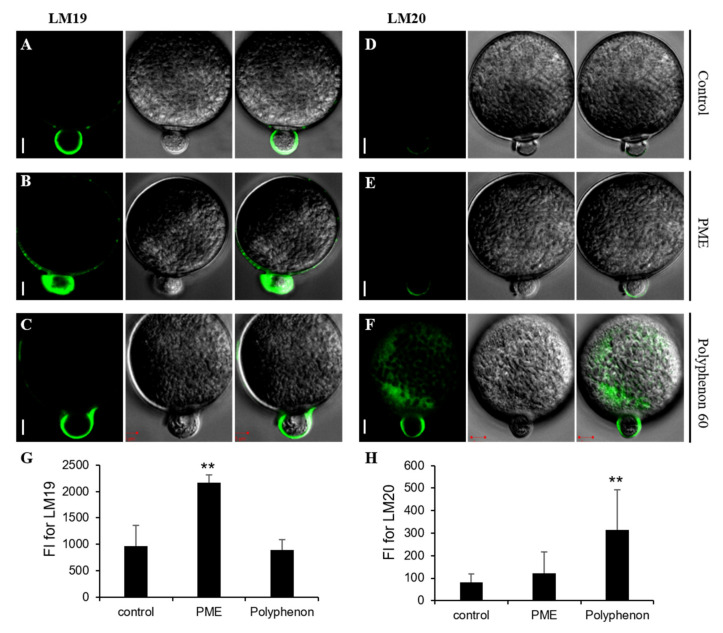
Immunodetection of pectins upon pollen germination. Pollen grains were probed with the monoclonal antibodies LM19 and LM20. LM19 is used to indicate pectins with a low level of methylesterification whereas LM20 binds to pectins with a high level of methylesterification. (**A**) Control: LM19-labeling signals upon pollen germination. (**B**) LM19-labeling signals of PME-treated pollen grain. (**C**) LM19-labeling signals of the Polyphenon 60-treated pollen grain. (**D**) Control: LM20-labeling signals upon pollen germination. (**E**) LM20-labeling signals of the PME-treated pollen grain. (**F**) LM20-labeling signals of the Polyphenon 60-treated pollen grain. Moderate concentration of PME (2 U/mL) was added onto the germination medium. (**G**) Quantification analysis of LM19 labeling. (**H**) Quantification analysis of LM20 labeling. FI, fluorescence intensity. All error bars represent SD of at least twenty independent experiments. Student’s *t* test: ** *p* < 0.01. Scale bars = 5 µm.

**Figure 3:**
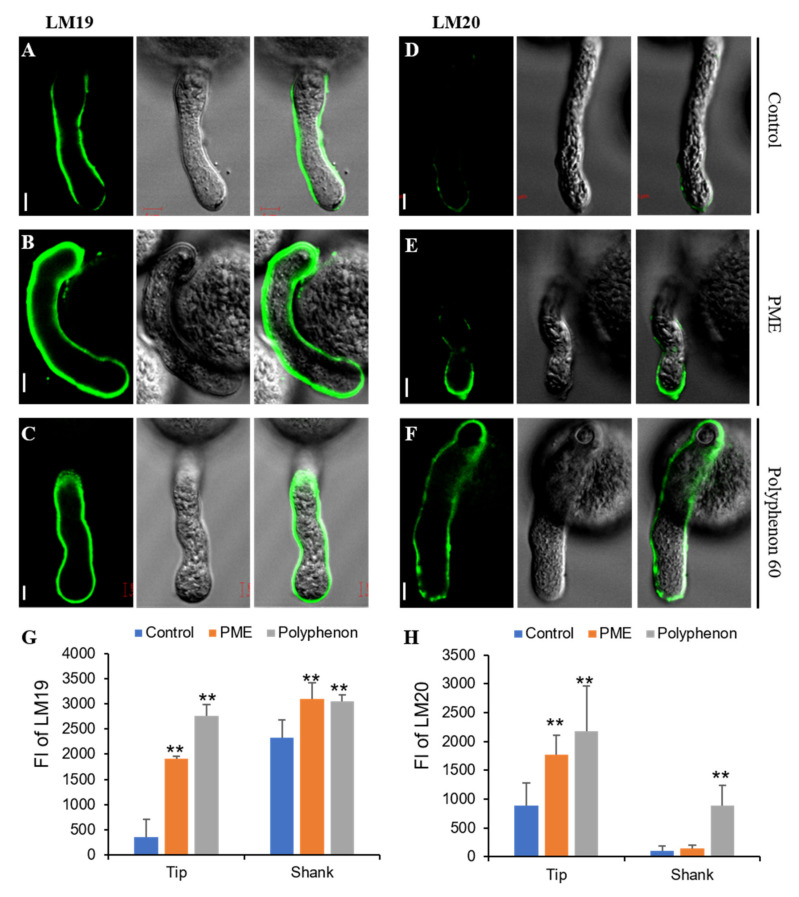
Immunodetection of pectins during pollen germination. Pollen tubes were probed with the monoclonal antibodies LM19 and LM20. LM19 is used to indicate pectins with a low level of methylesterification whereas LM20 binds to pectins with a high level of methylesterification. (**A**) Control: fluorescent signals of dimethyl-esterified pectin. (**B**) Fluorescent signals of dimethyl-esterified pectin of the PME-treated pollen tube. (**C**) Fluorescent signals of the dimethyl-esterified pectin of Polyphenon 60-treated pollen tube. (**D**) Control: fluorescent signals of methyl-esterified pectin. (**E**) Fluorescent signals of methyl-esterified pectin of the PME-treated pollen tube. (**F**) Fluorescent signals of methyl-esterified pectin of the Polyphenon 60-treated pollen tube. Moderate concentration of PME (2·U/mL) was added onto the germination medium. (**G**) Quantification analysis of fluorescent signals of dimethyl-esterified pectin on the pollen tube tip and shank. (**H**) Quantification analysis of fluorescent signals of the methyl-esterified pectin on the pollen tube tip and shank. FI, fluorescence intensity. All error bars represent SD of at least twenty independent experiments. Student’s *t* test: ** *p* < 0.01. Scale bars = 5 µm.

To further verify that PME and Polyphenon 60 treatments indeed lead to alteration of HGA deposition on pollen tubes, immunofluorescence assays using two pectin specific antibodies were performed. LM19 is used to indicate pectins with a low level of methylesterification whereas LM20 binds to pectins with a high level of methylesterification. Upon germination of rice pollen grains, a strong fluorescent signal by LM19 was observed in whole cell walls of a germinating tube, while weak fluorescent signals of the LM20 were observed at the apex of pollen tube (Figure 2A,D). During pollen tube elongation, LM19 signals were strongly observed in both the shank and the lateral region and weak signals was detected in the apical pollen tube cell wall (Figure 3A). In contrast, fluorescent signals of the LM20 immunolabeling were weakly observed at both the flanks of apex and the tip region (Figure 3D). These results are in accordance with dicot species in which highly methylated pectins are predominantly found in the pollen tube cell wall at the apex, while pectins with a low degree of methylesterification are present in the lateral region and the shank during pollen elongation. In order to directly observe the alteration of pectin deposition upon germination and during pollen tube elongation in the presence of PME or Polyphenon 60, a moderate concentration of each (2 U/mL PME and 1 mg/mL Polyphenon 60) was added onto the germination medium since an exposure at higher concentration resulted in the cessation of pollen tube growth. Exogenous PME treatment caused significantly increased fluorescent signals of the LM19 labeling in the whole cell walls of pollen tube upon germination (Figure 2B,G). LM20-labeled pectins of PME-treated pollen grains were weakly detected at the tip upon germination (Figure 2E,H). The deposition pattern of pectins labeled by LM20 upon germination was indistinguishable between the control and the PME-treated pollens (Figure 2D,E,H). Upon germination, Polyphenol 60-treated pollen showed similar LM19 labeling patterns to the control (Figure 2C,G). In Polyphenon 60-treated pollen grains, LM20-labeled signals were much stronger upon germination than the control (Figure 2F,H). PME treatment during pollen germination resulted in strong fluorescence of LM19 in the entire cell walls including the tip of pollen tube (Figure 3B,G). Similarly, a wider distribution of LM20-labeled pectin compared to the control was observed in the PME-treated pollen tube (Figure 3E,H). As expected, LM20 signals were observed in the whole cell wall of pollen tube by the Polyphenon 60 treatment (Figure 3F,H). Interestingly, similar to the PME-treated pollen tube, fluorescence signals of LM19 were detected in the entire cell wall in the Polyphenon 60-treated pollen tube (Figure 3C,G). These results suggest that Polyphenon 60 not only inhibits PMEs, but also exerts its unknown effect on pectin modification. Altogether, our immunodetection assay indicated that the distribution patterns of low- and high-methylesterified pectins upon germination and during tube elongation were disrupted by exogenous application of PME and Polyphenon 60.

### 2.3. Identification and In Silico Characterization of Rice Pollen-Specific PME and PMEI Genes

**Figure 4:**
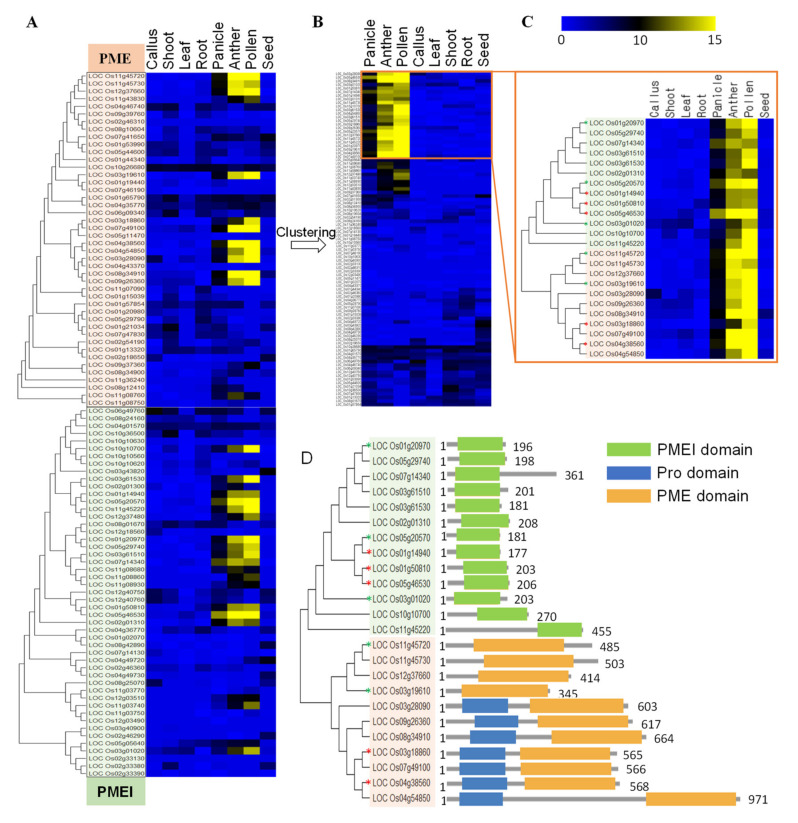
Meta-expression analysis and genome-wide identification of pollen-specific PMEs and PMEIs in rice. (**A**) Phylogenomic analysis and Heat map expression of PME and PMEI encoded in rice genome. (**B**) Clustering analysis by the HCL method/Euclidean distance/average linkage. (**C**) The focused heat map of pollen-preferred rice PME and PMEI genes. (**D**) Domain analysis of pollen-specific PME and PMEI proteins. The color of the cell in heat map represents transcript abundance. Blue colored boxes denote a low level of expression, and yellow colored boxes denote a high level of expression. Whole PME and PMEI genes in rice were analyzed for phylogenetic relationship and meta-expression.

**Figure 5:**
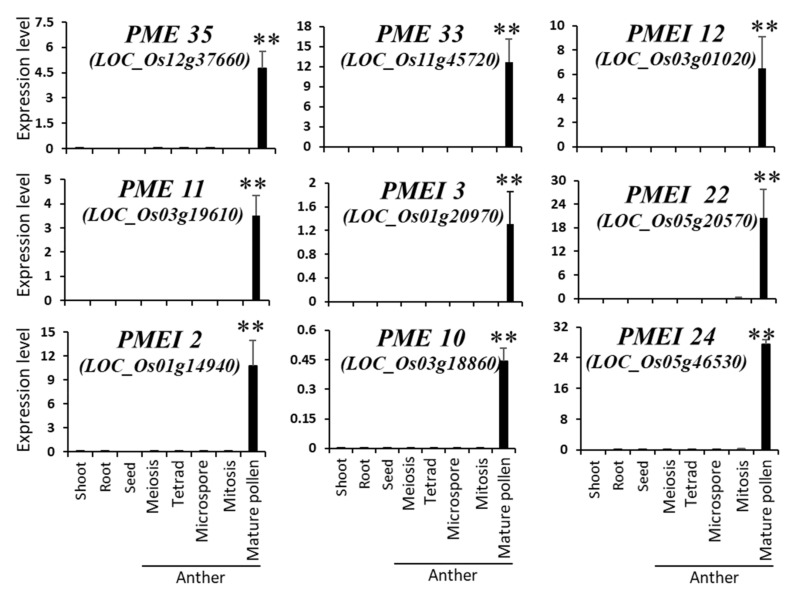
qRT-PCR examination. Validation of meta-expression patterns of pollen-expressed *OsPME*s and *OsPMEI*s using qRT-PCR. *OsUbi1* was used for internal control. ** *p*-value <0.01, based on *t*-tests.

Previously, our group reported a meta-expression analysis and genome-wide identification of pollen-specific genes in rice by utilizing the publicly available Affymetrix rice microarray data sets (NCBI GEO; http://www.ncbi.nlm.nih.gov/geo/) [27]. From these datasets, we found 11 *PME* and 13 *PMEI* genes, which were expressed during the middle and late stages of pollen development (Figure 4A–C and Tables S4 and S5). Among them, our group has previously confirmed the pollen-specific expression of *Os11g45730*, *Os07g14340,* and *Os05g46530* by the *β-glucuronidase* (*GUS*) reporter gene system [27]. We used qRT-PCR to validate their expression in rice pollen and found, in accordance with the microarray analysis, four *PME*s and five *PMEI*s were specifically expressed in anthers containing mature pollen grains (Figure 5). Based on the previous literatures [7,20], we named rice pollen-specific *PME*/*PMEI* genes: *OsPME10* (*Os03g18860*), *OsPME11* (*Os03g19610*), *OsPME12* (*Os03g28090*), *OsPME14* (*Os04g38560*), *OsPME16* (*Os04g54850*), *OsPME23* (*Os07g49100*), *OsPME26* (*Os08g34910*), *OsPME27* (*Os09g26360*), *OsPME33* (*Os11g45720*), *OsPME34* (*Os11g45730*), *OsPME35* (*Os12g37660*), *OsPMEI2* (*Os01g14940*), *OsPMEI3* (*Os01g20970*), *OsPMEI4* (*Os01g50810*), *OsPMEI6* (*Os02g01310*), *OsPMEI12* (*Os03g01020*), *OsPMEI15* (*Os03g61510*), *OsPMEI16* (*Os03g61530*), *OsPMEI22* (*Os05g20570*), *OsPMEI23* (*Os05g29740*), *OsPMEI24* (*Os05g46530*), *OsPMEI27* (*Os07g14340*), *OsPMEI35* (*Os10g10700*), and *OsPMEI48* (*Os11g45220*). An *in silico* analysis revealed that seven out of 11 *PME* genes contained an N-terminal pro region with moderate homology to PMEI proteins, and they are grouped into type 1 PMEs (Table S4). The domain structure of each PME and PMEI are depicted in Figure 4D. Similar to *Arabidopsis* type 1 PMEs, five out of seven rice pollen-specific type 1 PME possessed at least one of the conserved basic tetrad motifs (putative cleavage sites) at the N-terminal (Figure S1).

### 2.4. Enzyme Activity Assay Using the Tobacco Infiltration System

Full-length cDNA of two *PME* and three *PMEI* genes was successfully cloned from rice pollen cDNA and was delivered into overexpression vector for an enzyme assay. Since it can be difficult to produce enzymatically active recombinant PME and PMEI proteins in typical expression systems, we transiently expressed the aforementioned genes in *Nicotiana benthamiana* leaves under the control of the constitutive CaMV 35S promoter. In order to minimize the effects of both the endogenous tobacco PME and PMEI isoforms between different leaves, the respective constructs were injected into one half of the leaf, and the control construct was injected into the other half. After 48 h, crude protein extracts from each leaf half were extracted and PME and PMEI activities were measured in a coupled enzymatic assay.

**Figure 6:**
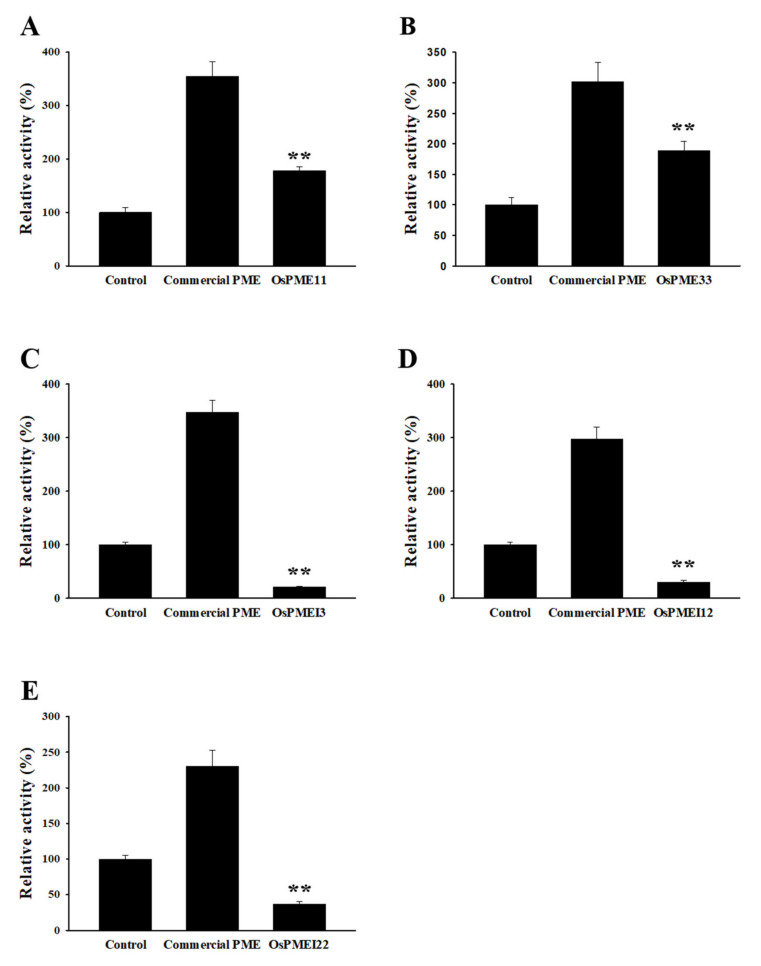
Enzyme activity assay of rice pollen-specific PMEs and PMEIs. Commercial PME (Sigma Aldrich, Cat No. P5400) was included in all reactions as a positive control. The empty vector was used as a control. The activity of commercial PME and PME/PMEI was expressed as a percentage of that of the control (empty vector). (**A**) PME11. (**B**) PME33. (**C**) PMEI3. (**D**) PMEI12. (**E**) PMEI22. Error bars represent the SE of three biological replicates. ** *p*-value <0.0s1, based on *t*-tests.

As a positive control, a commercial PME protein was included in all reactions. The mixtures were incubated for 30 min and then the amount of NADH was measured. As shown in Figure 6, transient expression of both PME11 and PME33 led to a 60% increase in PME activity in 30 min (Figure 6A,B); transient expression of PMEI3, 12 and 22 resulted in a reduction of PME activity (Figure 6C–E). Therefore, our biochemical characterization of rice pollen-specific PMEs and PMEIs indirectly demonstrated that they possess PME and PMEI activity, respectively.

### 2.5. Subcellular Localization of Rice Pollen Specific PMEs and PMEIs

**Figure 7:**
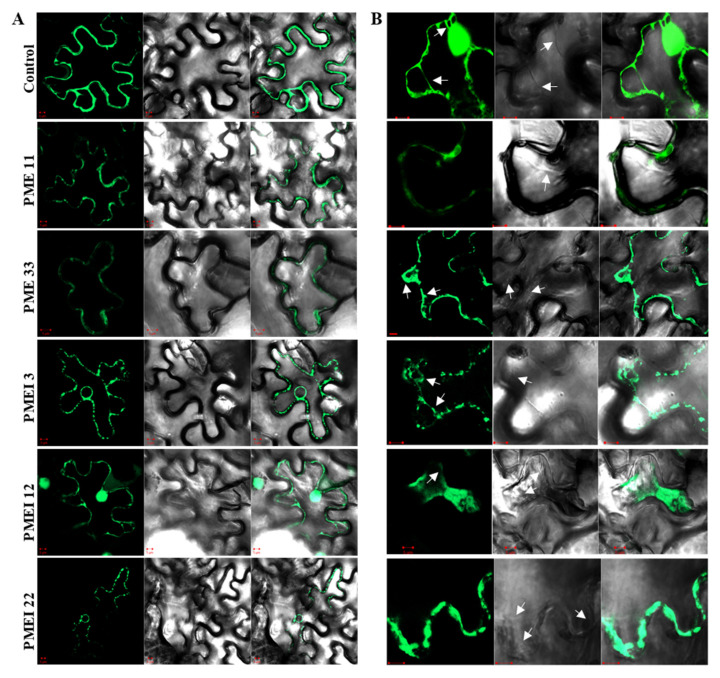
Subcellular localization of green fluorescent protein (GFP)-tagged OsPMEs and OsPMEIs in tobacco epidermal cells using confocal fluorescence microscopy. (**A**) Control-GFP, and five pollen-specific OsPME and OsPMEI signals were observed in tobacco epidermal cells. The bright image (left panel), the corresponding fluorescent signals (center panel), and a merged image (right panel). (**B**) Plasmolyzed tobacco epidermal cells. Cells were treated with 1 M NaCl, and arrows indicate the plasmolyzed Hechtian strands. The order of picture is the same as in A. Scale bars = 5 µm.

Considering that heavily methyl-esterified HGAs can be transported into cell walls, wherein they are subsequently demethyl-esterified, it is assumed that PME and PMEI proteins exert their enzymatic activities outside of the plasma membrane. Using the web tool (Signal P), the majority of rice pollen-specific PME/PMEI are predicted to be localized extracellularly (Tables S4 and S5). To confirm the subcellular localization of rice pollen specific PMEs and PMEIs, genes fused to the green fluorescent protein (GFP) were transiently expressed in tobacco leaves. The confocal analysis showed that the signals of PME11, PME33, and PMEI22 were observed in either the plasma membrane or apoplast (Figure 7A). In order to determine if they are targeted to the cell wall, the infiltrated leaves were plasmolyzed in 1 M NaCl (Figure 7B). Fluorescent signals of the control (35S-GFP) after plasmolysis were mainly observed in the cytoplasm. It was found that PME11 was clearly localized to the cell wall whereas the PME33-GFP and PMEI22-GFP fluorescence signal were observed in the plasma membrane after plasmolysis. The fluorescent signals of PMEI3-GFP and PMEI12-GFP were mainly found in the cytoplasm after plasmolysis.

## 3. Discussion

### 3.1. Fine-Tuning of PME and PMEI Activities is Required for the Proper Development of Pollen Tubes

Several lines of evidence have revealed that chemical modification of HGAs by PMEs and PMEIs cause profound changes in their mechanical properties and play a crucial role in a range of developmental processes [28,29,30,31]. Although it can be difficult to elucidate the microdomain structure of HGAs in elongating cells, immunolabeling experiments using pectin-specific antibodies showed that the fine-tuning of methyl-esterification on HGAs in pollen cell walls is required for proper pollen germination [16,19]. HGAs located in cell walls at the tip of the growing pollen tube are heavily methyl-esterified, while those far away they are relatively demethyl-esterified. Using LM19 and LM20 antibodies, the distribution of pectins along pollen tube cell walls was found to be similar to dicot plants (Figure 3A,D). Strong fluorescent signals of LM19 were only observed in the lateral region and shank, whereas LM20 signals were only detected at the tip region of pollen tube. Thus, it is expected that the enzymatic activities of PMEs located at the pollen tip would be inhibited by PMEIs. By contrast, PME-mediated methyl-esterification of HGA actively occurred in both the lateral region and the shank of pollen tube cell wall, possibly due to the absence of PMEI. In accordance with data pertaining to dicot plants, our findings confirmed that even moderate concentration of exogenous PME treatment severely affected pollen tube germination and growth (Figure 1), suggesting that a high degree of methyl-esterification is required for cellular expansion at the pollen tube tip. Previous studies reported that pectinase treatment on the in vitro pollen germination medium of *Solanum chacoense* induced pollen grain bursting [19]. Pectinase treatment is supposed to cause cell wall softening, thereby lowering the cell wall’s resistance. Interestingly, pollen tube bursting was also observed at the highest concentration of exogenous PME treatment (Figure 1G). Reduced levels of methyl-esterification by PME results in the increased cell wall stiffness. Thus, PME treatment turned the entire cell wall of pollen tubes to rigid structure. One possible explanation for PME-induced cell bursting we observed is that hyper-demethylesterified by exogenous PME HGA (as evidence by LM19 immunolabeling of PME-treated pollen grains, Figure 2B and Figure 3B) become a target for pectin-degrading enzymes, such as polygalacturonases [10]. Thus, the cell wall matrix of pollen tubes might be compromised and disintegrated. Furthermore, the addition of the PME-inhibiting catechin extract (Polyphenon 60) to an in vitro pollen germination medium at the moderate concentration led to a reduction in pollen tube germination and growth. At the high concentration, pollen tube burst was frequently observed (Figure 1L,M). This is consistent with previous observations that application of a recombinant protein ZmPMEI1 induces pollen tube bursting in a concentration dependent manner [32]. Using pectin specific monoclonal antibodies, we confirmed that the proper distribution of pectin along pollen tube cell walls was disrupted by either PME or the Polyphenon 60 treatment in the germination medium (Figure 2 and Figure 3). The entire cell walls of pollen tubes in the presence of exogenous PME deposited pectins with a low degree of methyl-esterification as revealed by the LM19 antibody. Interestingly, although it was expected that pectins with a high level of methylesterification would be deposited in the tube walls by the Polyphenon 60 treatment, intensive fluorescent signals of LM19 in the entire cell wall of pollen tube including at the tip region were detected (Figure 3C,G). The most plausible reason for the discrepancy may lie in the fact that Polyphenon 60 could not only inhibit PMEs, but also exerts the unknown effect on pectin modification. External application of rice pollen-specific PMEI proteins instead of Polyphenon 60 will undoubtedly reveal the importance of the degree of methyl-esterification of HGAs during pollen growth. Taken together, these findings suggest that the conversion of methyl-esterified HGAs into the demethyl-esterified HGAs, or vice versa, is essential in pollen tube growth and elongation for rice.

### 3.2. Several PME and PMEI Genes are Specifically Expressed in the Late Stage of Pollen Development in Rice

Eleven PMEs and 13 PMEIs were identified to be specifically expressed in pollen development in rice. An *in silico* domain structure analysis showed that seven PMEs belonged to type 1 (with pro-region) PME (Table S2). All PME genes were predicted to contain one or two conserved basic tetrad motifs, known to be a cleavage site in the Golgi apparatus of *Arabidopsis* [16]. Seven pollen-specific PMEs out of 13 contained a PMEI domain and cleavage sites at the N terminal, thereby grouping them into a type 1 PME. In *Arabidopsis*, biochemical characterization of VGD1 (group 1 PME) showed that cleavage of the N-terminal pro region by a subtilisin-like protease occurred outside of the cell [16]. Thus, it is proposed that enzymatic activities of group 1 PMEs are prevented in the Golgi apparatus, so as not to act prematurely on methyl-esterified HGAs. Since several rice pollen-specific PMEs have identical domains (basic motifs) in the N terminal, it would be interesting to experimentally determine if a similar post-translational modification occurs in monocot plants. Our qRT-PCR showed that these genes are predominantly expressed during the late stage of pollen development, yet their functional roles remain to be determined. Our preliminary screening of rice T-DNA lines found that some T-DNA lines with an insertion in either PME or PMEI gene displayed an abnormal segregation ratio (no homozygous progeny, unpublished data), leaving the function of various isozymes open for further investigation. Several studies have reported that mutation of pollen-specific PMEs in *Arabidopsis* caused abnormal pollen development [11,22,23,33]. Furthermore, other pectin remodeling genes including polygalacturonase and pectin acetylesterases have been shown to be involved in pollen development and pollen tube growth, indicating that coordinated actions of pectin modifying genes play a crucial role of the normal pollen development [34,35]. Future studies are needed to demonstrate the exact function of pollen-specific PMEs and PMEIs in rice.

### 3.3. Pollen-Specific PMEs and PMEIs Possess Unique Enzymatic Activities in Tobacco Leaves

Our demonstration that some of the pollen-specific PMEs and PMEIs that possess its unique enzymatic activities provide indirect biochemical evidence that such genes are functional during pollen development. Definitive proof that those genes exert its enzymatic activity would depend on demonstration of its biochemical activity using a recombinant protein. Future research on the optimization of heterologous expression of PME/PMEI is required. It should be noted that three pollen-specific PMEIs displayed strong PME inhibitory activities in tobacco leaves, indicating that rice PMEIs also inhibit endogenous tobacco PMEs. Similar results were also reported by AtPMEI2, and thus, it is possible that some PMEIs are capable of inhibiting more than one PME [16]. A simple explanation for this observation is that PMEI specificity is mainly determined by spatio-temporal distribution rather than its structure-based affinity to its partner PMEs. Our subcellular localization experiment using tobacco epidermal cells showed that PME11 appears to be targeted to the cell wall. PME33 and PMEI22 were localized to the plasma membrane whereas PMEI3 and PMEI12 appeared to be targeted to the cytoplasm after plasmolysis. A colocalization assay using propidium iodide (cell wall), FM4-64 (plasma membrane), and cytoplasmic markers would be needed to provide conclusive results on subcellular localization.

In conclusion, our findings of the importance of proper deposition of different levels of methylesterified HGA in the pollen tube cell walls and the presence of pollen-specific PMEs and PMEIs provides an important insight for further studies of pollen tube growth in rice.

## 4. Materials and Methods

### 4.1. Plant Growth and In Vitro Pollen Germination

Rice (*O. sativa* cv. Dongjin) seeds (Kyung Hee University, Yongin, Korea) were sterilized and germinated on Murashige and Skoog (MS) media (M0222, Duchefa Biochemie, Harlem, The Netherlands) and 10-days-old seedlings were transferred in the growth chamber and grown under controlled conditions (28/25′c day/night, 12-h photoperiod, and 78% relative humidity). To test in vitro pollen germination, pollen grains collected at dehiscence were germinated on the liquid pollen germination medium, consisting of 20% sucrose, 10% PEG, 3 mM calcium nitrate, 40 mg/L boric acid, and 10 mg/L vitamin B1 [34]. After incubation at 28 °C for 15–30 min, pollen germination and tube shapes were observed with a SZX61 microscope (Olympus, Tokyo, Japan). Various concentrations of PME (P5400, Sigma, St. Louis, USA) and Polyphenon (P1204, Sigma, St. Louis, USA) were added into medium before germination. Orange peel PME stock solution (0.78 unit·µL^−1^) was prepared in phosphate buffer, pH 7.0, and Polyphenon 60 stock solution (50 or 500 mg·mL^−1^) was prepared in sodium phosphate, pH 7.5. Control samples were treated with the respective buffer only. At least 200 pollen grains in each sample were examined for the germination ratio, and the data represent the average ± SD of three independent experiments.

### 4.2. Immunolocalization of Pollen Tubes

The immunolabeling method was performed according to the previously described protocol using *Arabidopsis* with modification [34]. Rice pollens were fixed by 5% (*v*/*v*) paraformaldehyde in the liquid germination medium for 30 min and quenched by the addition of 50 mM ammonium chloride in germination medium. The pollen was washed three times with the pollen germination medium and blocked for 1 h in 3% BSA (bovine serum albumin) in the germination medium. After washing, pollens were incubated 90 min at room temperature with a 1:200 dilution of the Rat IgM primary antibody against methylesterified (LM20) or de-esterified HGA (LM19; Plant Probes, UK). After washing three times with 3% BSA, the samples were incubated with 1:200 anti-rat IgG conjugated to FITC (Sigma-Aldrich) diluted in 3% (*w*/*v*) milk in the germination medium. The pollen was washed three times with the germination medium and viewed using a confocal laser scanning microscope (Zeiss LSM 510, Jena, Germany) with spectral settings of 535 nm for emission and 491 nm for excitation. LM19 and LM20 labeled images were collected with a ×40 PLAN objective by 190 µm of pinhole setting. The relative amounts of de-esterified and methylesterified pectin in pollen tubes were quantified by measuring the mean gray value using ImageJ software (http://rsbweb.nih.gov/ij/, version 1.52). Pixel intensity was measured along the periphery of each pollen tube, beginning from the pole (*n* >20 for each sample).

### 4.3. Genome-Wide Phylogenomic Analysis

The amino acid sequences and locus ID of rice PME and PMEI members were collected from the Rice Genome Annotation Project (RGAP, http://rice.plantbiology.msu.edu/) and Phytozome (https://phytozome.jgi.doe.gov/pz/portal.htmL; Table S1). Multiple amino acid sequences were aligned using ClustalW, and the phylogenetic tree was generated using MEGA 7.0.26 under neighbor-joining methods. Signal P version 5.0 was used online (www.cbs.dtu.dk/services/SignalP) to predict the presence of location of signal peptide and cleavage sites in amino acid sequences.

### 4.4. Tissue Expression Analysis

Meta-expression analysis of tissue-specific expression profiles were performed using the Multi Experiment Viewer (MeV, http://www.tm4.org/mev/) based on rice RNA-Seq data from NCBI SRA (https://www.ncbi.nlm.nih.gov/sra) and EMBL-EBI ArrayExpress (https://www.ebi.ac.uk/arrayexpress/). Clustering analysis was performed through HCL (hierarchical clustering) method. It also used Euclidean distance and the linkage method used the average linkage. We generated a heat map image based on log2 fold-change data and then integrated the heat map into the phylogenetic tree context. The generated heatmap were edited by Adobe illustrator. To examine gene expression in rice tissues, qRT-PCR was performed using various tissues, following the previous description using a real-time PCR machine. All of the qRT-PCR primers used are listed here (Table S2).

### 4.5. Vector Construction and Subcellular Localization Analysis

Mature anther cDNA was used to amplify the coding sequences (CDSs) of two PMEs and three PMEIs. The amplified CDSs were confirmed by sequencing and then cloned into the pGree vector fused with the C-terminal GFP. Sequences of all primers used in the study are listed in Table S3. The construcs were introduced into Nicotiana benthamiana leaves by Agrobacterium (strain *GV3101*)-mediated tranformation [36]. Two to three days after infiltration, GFP signals were observed with a confocal laser scanning microscope (Zeiss LSM 510, Jena, Germany) with spectral settings of 500–530 nm for emission and 488 nm for excitation.

### 4.6. PME and PMEI Activity Assay

Soluble protein extracts were prepared from *Nicotiana benthanmiana* leaves transformed with the control (empty vector), *OsPME11*, *OsPME33*, *OsPMEI3*, *OsPMEI12*, and *OsPMEI22*. Briefly, leaf tissues were homogenized with equal volumes (*w*/*v*) of extraction buffer (100 mM Tris-HCl, pH 7.5, 500 mM NaCl containing the protease inhibitor cocktail), followed by incubation at 4 °C for 120 min. The samples were spun down at 11,500 *g* at 4 °C for 20 min, and then the supernatant (protein extracts) was desalted using Microcon YM-10 centrifugal filter units (Millipore) into 10 mM Tris buffer, pH 7.7. Total protein concentration was determined by the BioRad protein assay kit with bovine serum albumin (BSA) as the standard. PME and PMEI activity assays were performed as previously reported [37]. For PMEI activity, 100 µg of soluble protein were premixed with a commercial PME (orange peel PME, P5400; Sigma-Aldrich) for 10 min and then the reaction mixture (a total volume of 1 mL) containing 0.4 mM NAD in 50 mM phosphate buffer, pH 7.5, 5% (*w*/*v*) pectin (P9135; Sigma), 0.35 unit formaldehyde dehydrogenase (F1879; Sigma), and 1.0 unit alcohol oxidase was added to the soluble protein/PME mixture. After incubation at 21 °C for 30 min, the reaction was terminated by heating in a boiling water bath. The PMEI activities to commercial PME were measured at 340 nm in a spectrophotometer. For PME activity, an identical experimental set up as the PMEI activity assay was performed with the exception of preincubation process. The commercial PME was included in all reaction as a positive control.

## Supplementary Materials

Supplementary materials can be found at https://www.mdpi.com/1422-0067/21/14/4840/s1. Table S1. Rice transcriptome data used in the study for genome-wide identification of pollen specific genes; Table S2. Primer sequences for qRT PCR; Table S3. Primer sequences used to isolate full-length cDNA for protein localization and enzyme activity; Table S4. Major features of pollen-specific PMEs; Table S5. Major features of pollen-specific PMEIs. Figure S1. Domain structure of group 1 PMEs studied in the study. Note the presence of at least one of cleavage site in the N-terminal. Four basic motifs (putative cleavage sites: RRLL, RRML, KLDL and RKLL) of the consensus sequence were found in rice group-1 PMEs.

## Abbreviations

HGA	Homogalacturonan
PME	Pectin methylesterase
PMEI	Pectin methylesterase inhibitor
GalUA	galacturonic acid
