# Supplementary material for: Physiological Importance of Pectin Modifying Genes During Rice Pollen Development
Source: Int J Mol Sci. 2020 Jul 8;21(14):4840. doi: 10.3390/ijms21144840 (PMC7402328; doi:10.3390/ijms21144840)
Supplement: Supplementary file 1 [file ijms-21-04840-s001.pdf]

# Supplementary Materials

**Table S1.** Rice transcriptome data used in the study for genome-wide identification of pollen specific genes.

| Run | Tissue_origin | Stage | Cultivar | Layout | Strategy | Source | Reads (Mbp) |
|---|---|---|---|---|---|---|---|
| DRR001029 | callus | | Japo_Nippon | Single | mRNA-Seq | 10.1093/gbe/evr111 | 831.5 |
| DRR001036 | callus | | Japo_Nippon | Single | mRNA-Seq | 10.1093/gbe/evr111 | 746.1 |
| DRR001043 | callus | | Japo_Nippon | Single | mRNA-Seq | 10.1093/gbe/evr111 | 506.9 |
| DRR001050 | callus | | Japo_Nippon | Single | mRNA-Seq | 10.1093/gbe/evr111 | 1600 |
| DRR001024 | leaf | 7 days before flowering to 7 days after flowering | Japo_Nippon | Single | mRNA-Seq | 10.1093/gbe/evr111 | 755 |
| DRR001031 | leaf | 7 days before flowering to 7 days after flowering | Japo_Nippon | Single | mRNA-Seq | 10.1093/gbe/evr111 | 525.8 |
| DRR001038 | leaf | 7 days before flowering to 7 days after flowering | Japo_Nippon | Single | mRNA-Seq | 10.1093/gbe/evr111 | 395.7 |
| DRR001045 | leaf | 7 days before flowering to 7 days after flowering | Japo_Nippon | Single | mRNA-Seq | 10.1093/gbe/evr111 | 1600 |
| DRR001025 | root | 7 days after germination | Japo_Nippon | Single | mRNA-Seq | 10.1093/gbe/evr111 | 687.1 |
| DRR001032 | root | 7 days after germination | Japo_Nippon | Single | mRNA-Seq | 10.1093/gbe/evr111 | 696.3 |
| DRR001039 | root | 7 days after germination | Japo_Nippon | Single | mRNA-Seq | 10.1093/gbe/evr111 | 392.7 |
| DRR001046 | root | 7 days after germination | Japo_Nippon | Single | mRNA-Seq | 10.1093/gbe/evr111 | 1600 |
| DRR001030 | seed | | Japo_Nippon | Single | mRNA-Seq | 10.1093/gbe/evr111 | 853.1 |
| DRR001037 | seed | | Japo_Nippon | Single | mRNA-Seq | 10.1093/gbe/evr111 | 734.3 |
| DRR001044 | seed | | Japo_Nippon | Single | mRNA-Seq | 10.1093/gbe/evr111 | 598.4 |
| DRR001051 | seed | | Japo_Nippon | Single | mRNA-Seq | 10.1093/gbe/evr111 | 1600 |
| DRR001026 | shoot | 7 days after germination | Japo_Nippon | Single | mRNA-Seq | 10.1093/gbe/evr111 | 574.9 |
| DRR001033 | shoot | 7 days after germination | Japo_Nippon | Single | mRNA-Seq | 10.1093/gbe/evr111 | 747.6 |
| DRR001040 | shoot | 7 days after germination | Japo_Nippon | Single | mRNA-Seq | 10.1093/gbe/evr111 | 414.5 |
| DRR001047 | shoot | 7 days after germination | Japo_Nippon | Single | mRNA-Seq | 10.1093/gbe/evr111 | 1600 |
| DRR001027 | pre-flowering panicle | 7 days before flowering | Japo_Nippon | Single | mRNA-Seq | 10.1093/gbe/evr111 | 809.5 |
| DRR001034 | pre-flowering panicle | 7 days before flowering | Japo_Nippon | Single | mRNA-Seq | 10.1093/gbe/evr111 | 744.2 |
| DRR001041 | pre-flowering panicle | 7 days before flowering | Japo_Nippon | Single | mRNA-Seq | 10.1093/gbe/evr111 | 430.4 |
| DRR001048 | pre-flowering panicle | 7 days before flowering | Japo_Nippon | Single | mRNA-Seq | 10.1093/gbe/evr111 | 755.6 |
| E-MTAB-7974 | Pollen | Mature pollen | Japo_DongJin | Paired | mRNA-Seq | | 5500 |
| E-MTAB-7974 | Pollen | Mature pollen | Japo_DongJin | Paired | mRNA-Seq | | 6000 |
| E-MTAB-7974 | Pollen | Mature pollen | Japo_DongJin | Paired | mRNA-Seq | | 5300 |
| E-MTAB-7974 | Anther | Mature Anther at anthesis time | Japo_DongJin | Paired | mRNA-Seq | | 5100 |
| E-MTAB-7974 | Anther | Mature Anther at anthesis time | Japo_DongJin | Paired | mRNA-Seq | | 4800 |
| E-MTAB-7974 | Anther | Mature Anther at anthesis time | Japo_DongJin | Paired | mRNA-Seq | | 4800 |

**Table S2.** Primer sequences for qRT PCR.

| Locus | Forward primer (5′→3′) | Reverse primer (5′→3′) |
|---|---|---|
| LOC_Os03g01020 | GCACGACGTGATCAACAATC | CGAGGCAGGTGATAGCCTTA |
| LOC_Os12g37660 | AAGGGGAAGTTCAGGACCAT | GTGAACGGGATCAACAGCTT |
| LOC_Os05g20570 | ATGAACACCCAGGACACCAT | TCTTCTCCAAGGGGTTGTTG |
| LOC_Os11g45720 | TCGGCACGCACTACATCTAT | TGCAGTTGAGTTCCTGGGTA |
| LOC_Os01g20970 | TCTACGACGACGACAGCATC | CCTCTGGCACATCTTCACCT |
| LOC_Os03g19610 | AGTTCAAGACCGTGCAGTCC | GGACTCGTGGTTGATTGAGG |
| LOC_Os01g14940 | GGCGGAGATATGCAAGAAGA | TACAGGCTGTCGCACAGGT |
| LOC_Os03g18860 | CTCAAGACGACGTTCGACAA | ATGTTCTCCGTCAGCTCCTG |
| LOC_Os05g46530 | ACCTGATCACCAACCCACTC | CAGCGTGTTGCACATCTTCT |

**Table S3.** Primer sequences used to isolate full-length cDNA for protein localization and enzyme activity.

| Locus | Forward primer (5′→3′) | Reverse primer (5′→3′) |
|---|---|---|
| LOC_Os03g01020 | GACGGTATCGATAAGCTT<br>ATGAGAATGAGCAAGGCCCT | AGGAATTCGATATCAAGCTT<br>TGGGTATTGGCTCTGTTG |
| LOC_Os05g20570 | GACGGTATCGATAAGCTT<br>ATGGGACCAGCCCACGGC | AGGAATTCGATATCAAGCTT<br>TATCATGCTGGCCAGTGTC |
| LOC_Os11g45720 | GACGGTATCGATAAGCTT<br>ATGGATCGTCCCAACCTC | AGGAATTCGATATCAAGCTT<br>TCTACTCGCCTTTGCCGG |
| LOC_Os01g20970 | GACGGTATCGATAAGCTT<br>ATGGCTTCCTCCCCGTAT | AGGAATTCGATATCAAGCTT<br>TTCTTCAGCGCTCGGTGA |
| LOC_Os03g19610 | GACGGTATCGATAAGCTT<br>ATGGCCCGGCCACGCCTC | AGGAATTCGATATCAAGCTT<br>GTAGTAGTACGCCGGGAG |

**Table S4.** Major features of pollen-specific PMEs.

| Gene Locus | Gene Name | PME Group Type | Mature Protein | | | Subcellular Localization Prediction Program (Signal P) |
|---|---|---|---|---|---|---|
| | | | Amino Acid | Kda | pI | |
| Os03g18860 | OsPME10 | 1 | 333 | 37.18 | 8.53 | Extracellular |
| Os03g19610 | OsPME11 | 2 | 345 | 38 | 8.87 | Extracellular, Chloroplast |
| Os03g28090 | OsPME12 | 1 | 338 | 36.49 | 7.53 | Extracellular, ER |
| Os04g38560 | OsPME14 | 1 | 323 | 36.2 | 9.58 | Extracellular, Vacuole |
| Os04g54850 | OsPME16 | 1 | 971 | 105.37 | 5.6 | Extracellular, Peroxisome |
| Os07g49100 | OsPME23 | 1 | 354 | 39.25 | 6.55 | Extracellular, Chloroplast |
| Os08g34910 | OsPME26 | 1 | 664 | 72.17 | 9.32 | Extracellular, Plasma membrane |
| Os09g26360 | OsPME27 | 1 | 360 | 39.22 | 8.07 | Extracellular, Chloroplast |
| Os11g45720 | OsPME33 | 2 | 485 | 50.91 | 8.5 | Extracellular, Chloroplast |
| Os11g45730 | OsPME34 | 2 | 503 | 53.5 | 9.21 | Extracellular, Chloroplast |
| Os12g37660 | OsPME35 | 2 | 414 | 44.32 | 9.39 | Extracellular, Chloroplast |

**Table S5.** Major features of pollen-specific PMEIs.

| Gene Locus | Gene Name | Subcellular Localization Prediction Program (Signal P) |
|---|---|---|
| Os01g14940 | OsPMEI2 | Extracellular |
| Os01g20970 | OsPMEI3 | Extracellular |
| Os01g50810 | OsPMEI4 | Extracellular |
| Os02g01310 | OsPMEI6 | Extracellular |
| Os03g01020 | OsPMEI12 | Extracellular |
| Os03g61510 | OsPMEI15 | Extracellular |
| Os03g61530 | OsPMEI16 | Extracellular |
| Os05g20570 | OsPMEI22 | Extracellular |
| Os05g29740 | OsPMEI23 | Extracellular |
| Os05g46530 | OsPMEI24 | Extracellular |
| Os07g14340 | OsPMEI27 | Extracellular |
| Os10g10700 | OsPMEI35 | Extracellular |
| Os11g45220 | OsPMEI48 | Extracellular |

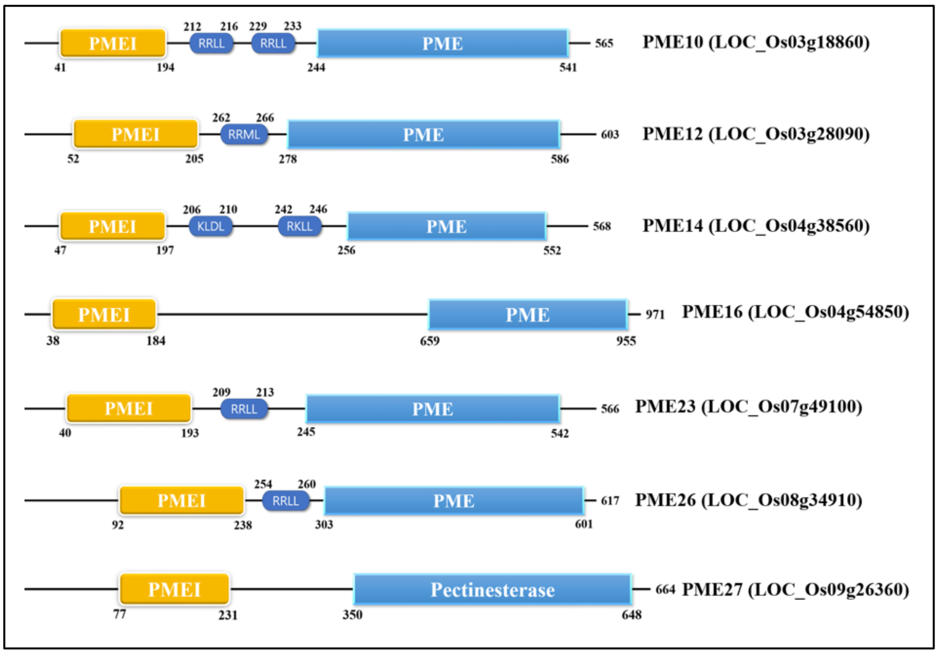


**Figure S1.** Domain structure of group 1 PMEs studied in the study. Note the presence of at least one of cleavage site in the N-terminal. Four basic motifs (putative cleavage sites: RRLL, RRML, KLDL and RKLL) of the consensus sequence were found in rice group-1 PMEs.
